# The POETIC (PrOcess Evaluation of Trials In Critical care) Framework: A Structured Approach for Designing and Conducting Process Evaluations in Critical Care Trials

**DOI:** 10.1097/CCE.0000000000001355

**Published:** 2025-12-02

**Authors:** Lydia M. Emerson, Daniel F. McAuley, Bronagh Blackwood, Mike Clarke

**Affiliations:** 1 The Medical School, Department of Health and Community Sciences, University of Exeter, Exeter, United Kingdom.; 2 Wellcome Wolfson Institute for Experimental Medicine, School of Medicine, Dentistry and Biomedical Sciences, Queen’s University Belfast, Belfast, United Kingdom.; 3 Centre for Public Health, School of Medicine, Dentistry and Biomedical Sciences, Queen’s University Belfast, Belfast, United Kingdom.

**Keywords:** critical care, health services research, process evaluation, randomized controlled trials, research design

## Abstract

**BACKGROUND:**

A process evaluation systematically examines how an intervention is delivered, including activities, procedures, and contextual factors influencing implementation. Existing process evaluation frameworks were primarily developed for education or public health settings, and do not reflect the complexity of critical care trials, which often involve medical technologies, high-acuity patients, and multidisciplinary care in dynamic environments. This study aimed to develop a framework (the POETIC (PrOcess Evaluation of Trials In Critical care) framework) to guide researchers in designing and conducting process evaluations that capture delivery quality and contextual understanding specific to critical care settings.

**METHODS:**

Framework development began in 2015 and followed an iterative, multi-phase process. Phase 1 included structured literature reviews to identify a) existing process evaluation frameworks and dimensions, and b) critical care trials with embedded process evaluations. Both reviews were updated in 2025 to reflect POETIC’s usage and ensure continued relevance. Phase 2 involved expert consultations with trialists, clinicians, and methodologists to refine framework dimensions.

**RESULTS AND CONCLUSIONS:**

Four key process evaluation frameworks and two U.K.-based critical care trials informed initial development. The 2025 update identified five additional U.K. trials, four of which applied POETIC, supporting its relevance and applicability. Expert consensus identified five core dimensions:

• Context (Unit Culture, Organizational Structure, Resources, Usual Practice, Attitudes and Perceptions)

• Fidelity (extent to which the intervention is delivered as intended)

• Dose (amount of the intended intervention delivered and received)

*•* Reach (extent to which the target population is exposed to, or engages with, the intervention)

• Quality of Delivery (integrative measure of Fidelity, Dose, and Reach)

The framework includes recommended methods such as checklists, interviews, routine trial data, and observations. It was iteratively refined to enhance usability and adaptability and has since been applied in multiple U.K.-based perioperative and critical care trials, demonstrating its utility in U.K. ICU settings. The POETIC framework supports structured evaluation of delivery quality and context in critical care trials, improving trial interpretation and advancing intervention design, delivery, and real-world applicability. Distinctively, POETIC operationalizes ICU-specific Context sub-constructs and provides a prespecified composite Quality of Delivery index to link intervention delivery to outcomes.

**KEY POINTS**

**Question:** What are the key components and considerations in developing a structured, context-specific framework to guide the design and conduct of process evaluations within critical care trials?

**Findings:** A multi-phase methodology involving literature reviews and expert consensus was used to develop the POETIC (PrOcess Evaluation of Trials In Critical care) framework, comprising five core dimensions (Context, Fidelity, Dose, Reach, Quality of Delivery) that offer a systematic approach to process evaluations in critical care trials. POETIC has been applied or planned in eight U.K. trials to date, seven funded by the National Institute for Health and Care Research (NIHR), demonstrating feasibility and growing adoption in diverse trial settings.

**Meaning:** The POETIC framework enables structured evaluation of delivery quality and context in ICU trials, supporting both real-time implementation adaptations and clearer interpretation of trial outcomes.

## BACKGROUND TO FRAMEWORK DEVELOPMENT

Randomized controlled trials (RCTs) are considered the gold standard for evaluating causal relationships between interventions and outcomes in healthcare (1, 2). In critical care, where patient populations are complex, dynamic, and heterogeneous, RCTs have been extensively conducted with the aim of improving clinical care and patient outcomes (3). Despite their potential, many critical care RCTs fail to demonstrate beneficial effects, making it difficult to distinguish between a true lack of efficacy and challenges in intervention delivery within the trial setting (4–6). Variability in patient acuity, clinical decision-making, and ICU practices, together with organizational norms and resource constraints, can compromise fidelity and challenge interpretation (7–11).

A process evaluation systematically examines trial and intervention delivery alongside contextual factors that may influence outcomes (12, 13). Typically, it considers fidelity (whether the intervention was delivered as intended), dose (the amount delivered), and reach (the extent to which the target population received the intervention) (13). By integrating mixed methods data, process evaluation can identify factors affecting implementation and delivery, enabling refinement of interventions and optimization of trial design (14). It is particularly valuable in explaining whether trial outcomes reflect the intervention itself or its delivery, offering insights into broader transferability (15, 16).

Although well established in public health and education settings (17–19), process evaluation remains underutilized in critical care, where the complexity of interventions and high-acuity environments make understanding delivery and context essential for interpreting outcomes (20, 21). Recent increases in neutral trial findings have driven greater uptake of process evaluations, but methods have varied and often lacked a theoretical foundation, underscoring the need for a standardized framework tailored to critical care (22, 23).

Existing process evaluation frameworks are largely oriented toward more stable settings (13, 19), rather than the distinctive challenges of critical care, such as high staff and patient turnover, complex multidisciplinary decision-making, and reliance on advanced technologies (4, 7, 21, 24). A context-specific framework can address these issues by enabling assessment of delivery quality and contextual variation, supporting stakeholder engagement, reducing research waste, and ensuring that critical care innovations are both effective and sustainable (25–27).

This study presents the development of the POETIC (PrOcess Evaluation of Trials In Critical care) framework; a structured, evidence-informed tool designed specifically for evaluating the delivery of complex interventions in critical care trials. By systematically capturing both delivery quality and contextual influences, the framework enables researchers to understand outcome variation and improve trial interpretation.

## FRAMEWORK DEVELOPMENT PROCESS

The POETIC framework development was initiated in 2015 in response to growing recognition that outcome data alone often fail to explain the results of complex critical care trials. In these settings, interventions are shaped by diverse clinical practices, organizational factors, and rapidly changing patient conditions. Without insight into how interventions are delivered and influenced by their context, interpreting outcomes remains challenging, whether trials are neutral, inconclusive, or demonstrate benefit. For example, when a trial shows a positive effect, understanding the contextual and delivery factors that contributed to success is critical for planning wider implementation and ensuring that benefits can be reproduced in different settings. To address this, a structured, multi-phase process was undertaken, beginning with two comprehensive literature reviews and followed by expert consultation, to develop a framework that would support deeper understanding of trial results. A central aim was that the framework could support both retrospective analysis and real-time adaptation during trials, helping trial teams refine implementation strategies as the trial progresses. The framework was intentionally confined to process evaluation models to maintain focus on delivery during the trial phase; broader implementation science frameworks, although valuable for post-trial adoption and scale-up, were outside scope to avoid misalignment with POETIC’s objectives. POETIC should therefore be understood as a scaffold for trial-based process evaluation, which may be complemented by implementation frameworks when considering scale-up or as interpretive lenses for understanding wider adoption.

Two structured literature reviews were originally conducted in 2015 and 2016 as part of the framework development process and were subsequently updated in July 2025 to ensure the ongoing relevance of the POETIC framework. The first review aimed to systematically identify and appraise existing process evaluation frameworks or guidance, assessing their contexts, dimensions, and applicability to critical care clinical trials. A comprehensive search was performed in MEDLINE and Embase from database inception to October 1, 2015, supplemented by manual screening of reference lists, and repeated in July 2025 using the same strategy. Studies were eligible if they explicitly provided a process evaluation framework or detailed guidance on conducting such evaluations. Screening was conducted independently in duplicate with consensus resolution of disagreements. Data extraction was performed using a structured, piloted form capturing framework context, dimensions, critical care applicability, and reported strengths and limitations, with findings synthesized narratively.

The second review focused on critical care trials that had incorporated process evaluations. Searches covered MEDLINE and Embase from database inception to December 1, 2016, updated in July 2025, with additional manual screening of reference lists and key critical care journals. Eligible studies were those conducted in critical care settings that explicitly reported a process evaluation, including evaluation methods, frameworks, data collection strategies, and key findings. Only U.K.-based trials were included to ensure contextual consistency within the National Health Service (NHS), aligning the resulting framework with the operational realities of U.K. critical care environments. Screening and data extraction followed the same dual-reviewer procedures and used structured forms tailored to this review, capturing study design, process evaluation approach, findings, and reported strengths and limitations.

PRISMA flow diagrams, full search strategies, and data extraction forms are all provided in the **Supplementary Material** (https://links.lww.com/CCX/B578). Limitations include restriction to two databases, exclusion of grey literature, and a U.K.-only focus in the second review, which may affect generalisability.

Expert input was obtained through two complementary processes, consistent with the Standards for Reporting Qualitative Research (SRQR) guidance (28). First, ongoing consultation took place with the four coauthors: Professor of Research Methodology (M.C.), Professor of Intensive Care Medicine and Consultant Intensivist (D.M.), Professor of Critical Care and Nurse with expertise in process evaluation (B.B.), and the first author, a clinical trials methodologist with expertise in process evaluations in critical care (L.E.). These coauthors were purposively engaged for their complementary expertise spanning methodology, intensive care medicine, nursing, and process evaluation. Framework development and analysis were led by the first author (L.E.), with monthly structured discussions involving all coauthors to provide critique and iterative feedback. This ensured integration of methodological, clinical, and process evaluation perspectives, and enhanced trustworthiness by involving multiple team members in reviewing and synthesizing feedback to reduce reliance on a single analyst and maintain reflexivity in interpretation.

Second, a focused workshop was convened with senior academics selected for their direct experience of conducting process evaluations within clinical trials. Participants included B.B., L.E., a Professor of Critical Care, a Lecturer in Nursing with expertise in critical care and process evaluation, and a Professor of Medical Statistics with experience in large-scale trials and process evaluation. The workshop explored the use and perceived utility of existing process evaluation frameworks in practice and provided structured discussion of approaches for developing a composite quality of delivery measure. Feedback from this meeting informed both the refinement of the POETIC dimensions and the approach to operationalizing Quality of Delivery. Input was documented and thematically summarized by the study team to ensure consistency and transparency.

The resulting framework was specifically designed to address the unique complexities and challenges inherent in critical care clinical trials. It enables researchers to evaluate how context and delivery quality influence outcomes, thereby supporting improved interpretation, more effective intervention delivery, and better-informed future trial design. As one of the first structured tools tailored to this setting, POETIC fills a critical methodological gap in the design and conduct of process evaluations in critical care.

### Ethics Approval and Consent to Participate

Expert consultation undertaken during framework development did not involve human participants or identifiable personal data. In accordance with the Queen’s University Belfast Research Ethics Committee policy, this activity did not constitute human subjects research and was therefore exempt from ethics review. Consent to participate was not required.

## RESULTS

The first review identified four process evaluation frameworks published between 1999 and 2015: two identified directly from the literature search (13, 29), and two identified from searching reference lists (19, 30). These provided the foundation for defining key dimensions that would form the basis of a context-specific framework suitable for critical care trials. The 2025 update confirmed that no new process evaluation frameworks or guidance had been published in the intervening years, reaffirming the ongoing relevance and uniqueness of POETIC. Each framework provided detailed guidance on conducting process evaluations but there was considerable heterogeneity between them in terms of terminology, depth, and scope. Although distinct, substantial overlap existed among their dimensions, particularly around core concepts such as fidelity, dose, and reach. Despite these commonalities, inconsistency in how these dimensions were defined and labeled across process evaluation frameworks posed challenges for their application.

A 2016 review of process evaluations within critical care trials identified only two U.K. studies that explicitly reported such evaluations (31, 32). In the 2025 update, five additional U.K.-based critical care trials incorporating process evaluations were identified (33–37). Four of these used the POETIC framework: Biomarker-guided antibiotic stewardship in suspected ventilator-associated pneumonia (VAPrapid-2); Provision of Psychological Support to People in Intensive Care (POPPI), Sedation AND Weaning in CHildren (SANDWICH), and Alpha-2 agonists for sedation to produce Better outcomes from critical illness (A2B). These trials applied elements of POETIC during development or to validate and refine the framework in real-world ICU settings. In contrast, the “CoolCuddle” study was guided by Normalization Process Theory (38), which was used to evaluate how neonatal staff understood, engaged with, enacted, and appraised the intervention. The repeated use of POETIC across multiple trials not only provided iterative feedback for framework refinement but also demonstrates increasing recognition by researchers and funders of the value of structured process evaluation in critical care. The included studies demonstrated variability in the dimensions assessed, with no consistent use of a standardized framework. This limited and inconsistent application highlights the fragmented nature of process evaluation practice in critical care and underscores the lack of established methodological standards, making it difficult to draw generalizable conclusions about the effectiveness of trial implementation.

**Figure 1.**
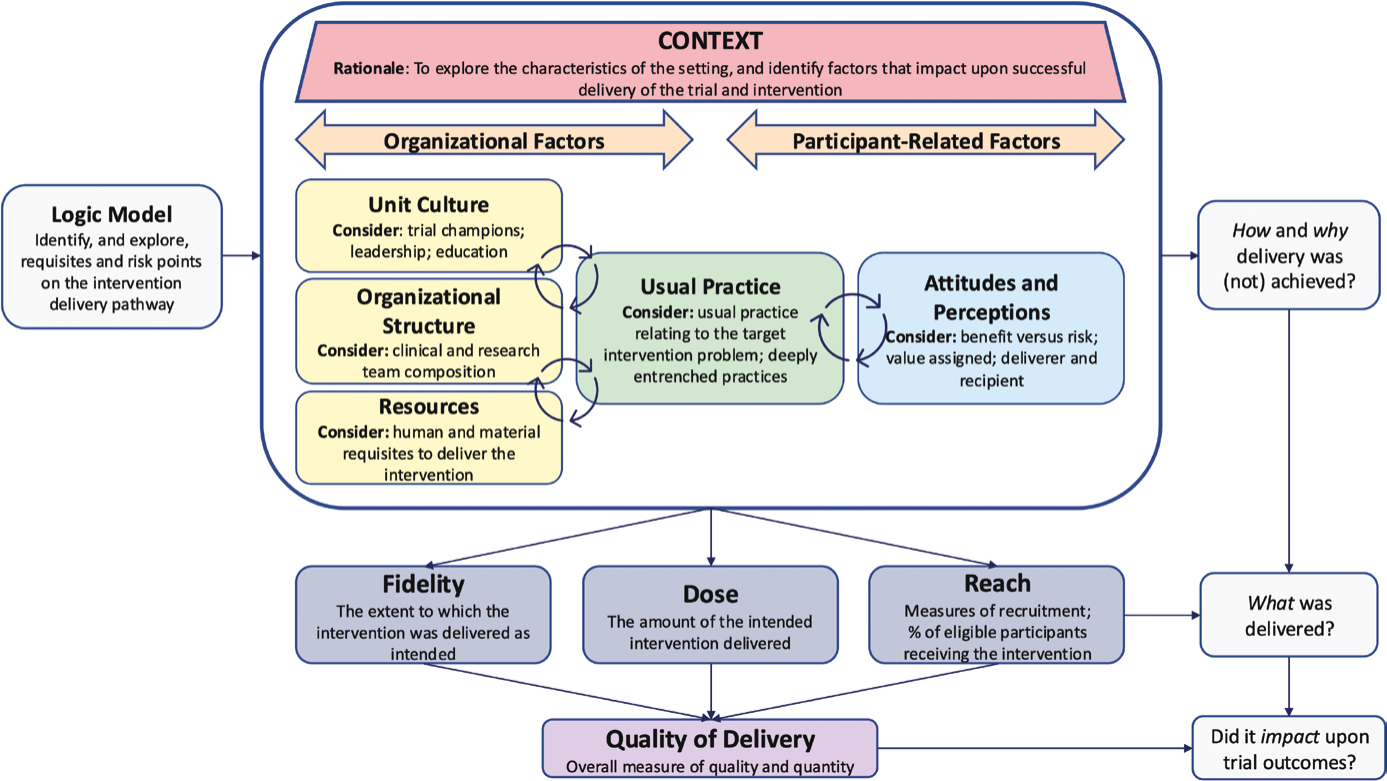
Schematic representation of the POETIC (PrOcess Evaluation of Trials In Critical care) framework. This figure presents the five core dimensions (Context, Fidelity, Dose, Reach, Quality of Delivery), their sub-constructs, and conceptual interrelationships within the framework for process evaluation in critical care trials. Context informs Fidelity, Dose, and Reach, which together contribute to overall Quality of Delivery.

To address the variability and overlap among existing process evaluation frameworks, the POETIC framework prioritizes clear, concise dimensions with standardized terminology. Based on a synthesis of existing process evaluation frameworks and critical care-specific needs, the POETIC framework comprises five core dimensions: Context, Fidelity, Dose, Reach, and Quality of Delivery. A schematic representation of the framework’s structure and the interrelationships among its dimensions is provided in **Figure 1**. Suggested methods and data sources for evaluating each dimension of the POETIC framework are outlined in **Supplementary Material**, **Table S1** (https://links.lww.com/CCX/B578). To further clarify positioning, **Table 1** maps the POETIC dimensions against the four frameworks identified in the reviews. This shows areas of alignment (particularly around fidelity, dose, reach, and context), as well as points of divergence, including POETIC’s explicit operationalization of ICU-specific contextual sub-constructs, addition of a composite Quality of Delivery dimension, and use of phased data collection.

**TABLE 1.** Mapping of the POETIC (PrOcess Evaluation of Trials In Critical care) Framework Dimensions Against Four Established Process Evaluation Frameworks

POETIC Dimension	Glasgow et al (1999)	Steckler and Linnan (2002)	Grant et al (2013)	Moore et al (2015)	Novel Contribution of POETIC
Context	Implied (adoption/maintenance)	Core element	Core element	Core element	Specifies ICU-relevant sub-constructs. These capture how local ICU conditions shape delivery and feasibility, making context explicit and operationalizable.
Fidelity	Explicit (implementation)	Core element	Explicit (delivery)	Explicit	Defined as the extent to which the intervention was delivered as intended. Adapted for ICU complexity, including variation across multidisciplinary teams, shift patterns, and high-acuity clinical pressures.
Dose	Implied (frequency/intensity under implementation)	Core element	Implied (delivery)	Explicit	Refers to the amount of intervention delivered and received. In ICU settings, this requires explicit consideration of time constraints, workload pressures, and rapid patient turnover that may limit intended exposure.
Reach	Core element	Recruitment/Reach	Recruitment/reach	Explicit	Captures the extent of exposure to the intervention within the eligible/target population. POETIC highlights ICU-specific challenges such as fluctuating eligibility, unpredictable admissions, and high patient turnover that affect representativeness.
QoD	Not explicit	Explicit focus on delivery quality	Not explicit	Implicit within fidelity/ mechanisms	An integrative dimension that combines Fidelity, Dose, and Reach. Reflects both the quantity and QoD, operationalized through a composite QoD index that enables systematic assessment of variation in delivery patterns (e.g., differences in amount, consistency, and reach) within and across trial sites.
Phased data collection	Not specified	Not specified	Not specified	Not specified	Unique to POETIC: introduces an explicit three-phase structure (Baseline, During-Trial, End-of-Trial). Enables both retrospective evaluation and real-time feedback to support adaptive trial conduct.

POETIC = PrOcess Evaluation of Trials In Critical care, QoD = Quality of Delivery.

This table highlights areas of alignment, divergence, and novel or ICU-specific contributions.

### Context: The Setting in Which the Intervention is Delivered

Context plays a pivotal role in implementation success and the generalizability of trial findings. POETIC identifies five sub-constructs of Context, each addressing different organizational and participant-related influences on intervention delivery:

a) Unit Culture: assesses the presence of trial champions, leadership behaviors, and internal communication or educational structures that influence team engagement.
b) Organizational Structure: examines the composition and roles of both clinical and research teams, including how staffing configurations support or constrain delivery.
c) Resources: evaluates the availability of human and material resources necessary to conduct the trial and deliver the intervention effectively.
d) Usual Practice: explores how care is typically delivered for the clinical problem targeted by the intervention, including the extent to which established routines or entrenched practices may support or conflict with the new approach.
e) Attitudes and Perceptions (novel dimension): introduced as a unique addition to POETIC, this dimension captures the beliefs, concerns, and perceived value of the intervention from both those delivering and receiving it. Unlike previous process evaluation frameworks, which often subsumed such influences under broader behavioral or contextual categories, POETIC recognizes Attitudes and Perceptions as a discrete and critical dimension. This is particularly important in ICU settings, where high acuity, interdisciplinary dynamics, and risk sensitivity shape perceptions of intervention worth, perceived benefit versus risk, and overall engagement. This dimension helps to explain how such perceptions affect both intervention fidelity and team behaviors throughout trial delivery.

#### Suggested Data Sources

a) Documentary analyses (e.g., policies and protocols).
b) Surveys and questionnaires.
c) Patient and family participant interviews and observations to capture experiences of care, perceptions of the intervention, and relational dynamics with staff.
d) Clinical staff interviews and focus groups to explore factors influencing successful trial and intervention implementation and delivery.
e) Observations during intervention delivery.
f) Analysis of reflective feedback to explore changes in perceptions over time.

### Fidelity: The Extent to Which the Intervention is Delivered as Intended

Fidelity was a consistent dimension across all process evaluation frameworks, though inconsistently labeled (e.g., “delivery” or “implementation”). Accurate Fidelity assessment is essential to understanding intervention effectiveness. Evaluation involves empirically assessing intervention delivery, identifying factors affecting protocol adherence, and documenting strategies for overcoming delivery challenges.

Fidelity assessments may use core component checklists, observer analyses, surveys, and interviews. For complex interventions, qualitative methods are essential to explore delivery variations and the skills and training of those implementing and delivering the intervention. The interplay between Fidelity and adaptations, as highlighted in the Medical Research Council (MRC) guidance, is particularly relevant in critical care, where non-adherence to strictly protocolized interventions is classified as “protocol deviation.” Acceptable adaptations (e.g., modifying vocabulary; using culturally appropriate images) can enhance intervention relevance, while risky adaptations (e.g., altering dose or theoretical approach) may compromise effectiveness.

#### Suggested Data Sources:

Fidelity assessment should suit the intervention design and balance cost, efficiency, and bias minimization. Common sources include:

a) Adherence checklists and surveys for quantitative data.
b) Case report forms documenting protocol deviations.
c) Observational data, field notes, and interviews for qualitative insights.

### Dose: The Amount of the Intended Intervention Delivered and Received

All process evaluation frameworks recognized Dose as a key source of variation. Evaluation involves measuring both Dose delivered (quantity provided) and Dose received (quantity received; participation and/or engagement).

#### Suggested Data Sources:

Dose assessment depends on intervention design and may involve:

a) Quantitative measures, e.g., session counts, participant attendance.
b) Training uptake as a proxy for intervention exposure.
c) Pre-defined minimum acceptable doses established during study design to interpret trial outcomes.

### Reach: The Extent to Which the Intended Target Population is Exposed to, or Engages With, the Intervention

Reach reflects both the proportion of intended recipients who were recruited and the broader success of engaging eligible participants. It captures critical factors related to accessibility, engagement, and representativeness, and provides insights into barriers to delivery and uptake, particularly useful in feasibility and pilot studies. Recruitment was initially considered as a standalone dimension but was ultimately refined and incorporated within Reach. This reflects an understanding that recruitment is a key mechanism through which Reach is operationalized, especially in trials involving complex critical care interventions.

#### Suggested Data Sources:

a) Screening and recruitment logs detailing eligibility, consent, and enrollment outcomes.
b) Interviews with research and clinical staff to identify factors influencing successful delivery, and recruitment dynamics.
c) Routine trial data to examine site-level variations in recruitment trends and performance.
d) Clinician reflections on perceived fit, acceptability, or risks associated with the intervention.

### Quality of Delivery: The Integrative Dimension

Although Fidelity, Dose, and Reach provide valuable insights individually, critical care trials require an overarching view of how these factors interact within a specific ICU environment. In the POETIC framework, this is captured under the dimension of Quality of Delivery. Guided by Steckler and Linnan (30), this term emphasizes not only technical adherence to intervention protocols but also the spirit and nature of delivery: how well the intervention was enacted in practice.

Quality of Delivery serves as a summative construct, integrating data on Fidelity, Dose, and Reach to provide an overall assessment of how consistently and effectively the intervention was delivered as a whole. This enables researchers to move beyond isolated measures (e.g., high Fidelity but limited Reach) and understand the collective influence of delivery on trial outcomes.

By conceptualizing quality of delivery as a distinct dimension, POETIC provides a practical mechanism for quantifying and comparing intervention delivery across sites, through composite or integrative analyses. This supports trial interpretation by distinguishing outcome variation due to genuine intervention effects from variation in delivery quality.

### Alignment With TIDieR/StaRI Reporting Principles

The POETIC framework is designed to support transparent reporting of intervention delivery in line with the Template for Intervention Description and Replication (TIDieR) and Standards for Reporting Implementation studies (StaRI) guidance (39, 40). Each of the five dimensions incorporates elements central to these reporting standards. Fidelity requires trialists to specify core intervention components and distinguish them from aspects where adaptation is permitted, ensuring clarity about what must be standardized across sites. Dose encompasses both intended schedules and actual exposure, capturing variation across patients, sites, or phases of care. Reach extends this by documenting who was exposed to the intervention and who was not, relative to the eligible population. Quality of Delivery integrates Fidelity, Dose, and Reach into an overall summary while also capturing how delivery was enacted in practice. Finally, Context captures the organizational and systemic conditions that shape feasibility and sustainability, aligning with StaRI’s emphasis on implementation strategies and local adaptation. Together, these elements ensure POETIC not only provides a framework for process evaluation but also enables trialists to report their interventions in ways that facilitate replication and interpretation. Operational tools to support this alignment will be detailed in the third article of this series.

### Phases of Process Data Collection

Three distinct phases of process data collection are proposed to ensure that contextual influences and delivery quality are systematically captured at key stages of a trial, enabling both formative feedback during conduct and explanatory insights at trial completion.

Baseline (Pre-Trial Data): Examine how the target intervention practice is usually managed and identify factors that may influence implementation, such as unit capacity and contextual conditions, before recruitment begins. Suggested methods include review of unit policies and protocols, surveys, and interviews with clinical and research staff.

Exploration (During-Trial Data): Identify factors influencing successful delivery mid-trial to refine implementation. This phase explores Fidelity, Dose, Reach, and evolving attitudes toward the intervention. Suggested methods include routine trial data review, interviews, and observations. Participants may include staff involved in trial and intervention delivery, patients, and families.

Clarification (End-of-Trial Data): Combine mixed methods data to explain during-trial findings and assess intervention delivery over time. Suggested methods include routine trial data review, interviews, and observations. Participants may include staff involved in trial and intervention delivery, patients, and families. Use Quality of Delivery index scores for exploratory analyses.

POETIC is intended as a scaffold to support the identification and structuring of process evaluation dimensions and content, rather than as prescriptive guidance on data analysis. The choice of analytic approach will depend on the intervention, trial design, and selected data collection methods. A key principle, however, is that analysis of process evaluation data should be completed before trial outcomes are known to evaluators, to minimize bias and maintain the explanatory integrity of findings.

### Distinguishing Between Trial and Intervention Processes

The POETIC framework explicitly distinguishes between processes related to trial delivery and those associated with intervention delivery. This distinction is essential for accurately identifying the source of implementation challenges and understanding their impact on trial outcomes. This distinction also plays a crucial role in helping researchers determine whether a lack of observed effect in a trial reflects a failure of the intervention itself, or issues in how it was implemented or delivered. Trial-related processes refer to activities required to conduct the study, such as site setup, recruitment, and data collection; while intervention-related processes encompass the delivery, uptake, and integration of the intervention within the trial setting. By separating these components, the framework ensures that impacting factors are appropriately attributed, avoiding misinterpretation of whether issues lie with the trial infrastructure or the intervention itself. This clarity enhances the accuracy of process evaluation findings and supports more targeted improvements in future trial design and delivery.

## DISCUSSION

The POETIC framework can provide important insights into factors influencing delivery of complex interventions in critical care trials, emphasizing systematic evaluation of key dimensions: Context, Fidelity, Dose, Reach, and Quality of Delivery. By synthesizing existing process evaluation frameworks, POETIC addresses inconsistencies in terminology and definitions, standardizing dimensions to improve usability and comparability in ICU research. Designed for the high-acuity, multidisciplinary nature of critical care, it underscores the importance of embedding process evaluation early in trial planning. Its innovation lies in integrating evaluation throughout trial design and conduct, rather than treating it as supplementary. This approach enables rapid feedback, timely resolution of challenges, and ultimately improves the interpretability, efficiency, and transparency of trial findings.

Transparent use and reporting of POETIC across future trials will also be vital. Doing so will help build shared knowledge around what works, where, and why; encouraging cumulative learning, reducing research waste, and strengthening critical care trial design. By enabling structured evaluation of delivery quality and contextual variation, POETIC helps ensure that trial outcomes, whether positive, negative, or neutral, are accurately interpreted. It will strengthen the evidentiary base for interventions in critical care and support more informed decisions around scale-up, modification, or de-implementation.

The updated reviews confirm both the sustained relevance and increased adoption of POETIC since its original development. Its use in additional trials supports the framework’s adaptability and rigor, although broader application in non-ICU settings will be needed to further test its transferability. Several dimensions of POETIC, such as Fidelity, Dose, Reach, and Quality of Delivery, are ICU-universal. However, contextual factors that shape their expression, such as staffing models, funding structures, and governance arrangements, reflect NHS-specific features and will require consideration and adaptation in health systems with different payment models, regulatory environments, or workforce configurations.

There are limitations. Methods for statistically linking process data with outcomes remain underdeveloped. Detailed guidance on the specification and analysis of composite Quality of Delivery scores, including worked examples and analytic strategies, will be provided in the third article of this series. As outlined, POETIC was deliberately confined to process evaluation models to maintain focus on delivery during the trial phase, with implementation frameworks remaining relevant primarily for post-trial translation. These may be used in tandem with POETIC, depending on whether evaluators wish to extend interpretation or address questions of scale-up. A further limitation is that both the review and the resulting framework were confined to U.K. trials. While this ensured contextual consistency within the NHS, validation in non-U.K. settings will be required to determine broader applicability (14, 27).

Although originally developed for critical care, POETIC is now being used in two ongoing NIHR-funded non-ICU trials: CompreHensive geriatric Assessment for oldeR people with hearT failure and frailty (CHART, NIHR155936); and Sugammadex for preventIoN oF pOst-operative pulmoNary complIcAtions (SINFONIA, NIHR 133056). It has been integrated into the design of two upcoming NIHR-funded multicenter ICU trials (one pediatric), and is also being used in a non-ICU implementation study examining broader roll-out. These applications will test whether the framework’s core dimensions are transferable beyond adult ICU trials or require adaptation. Early indications suggest good performance across contexts, though domains such as unit culture or risk perception may need refinement in different environments. Findings from these evaluations, including methodological insights and adaptations, will be reported in detail in the second article of this series.

The COVID-19 pandemic highlighted missed opportunities for methodological innovation. Despite rapid delivery of large-scale U.K. critical care trials such as RECOVERY (41), none embedded a process evaluation. This absence may reflect concerns about delays or recruitment impact (42, 43). POETIC’s phased structure demonstrates how a rapid-cycle module could provide timely insights into Quality of Delivery and Context without compromising trial pace, offering a feasible approach during surge conditions. Operational guidance for such a module, along with reusable tools such as fidelity checklists, dose and reach logs, exemplar interview guides, and templates, will be provided in the third article of this series, designed as a practical toolkit.

Ultimately, POETIC helps bridge the gap between evidence generation and implementation. By supporting clearer interpretation of outcomes, more consistent evaluation of delivery, and scalable application, the framework enhances both trial success and the uptake of effective interventions. Its standardized structure promotes comparability and cumulative learning across ICU studies, providing a foundation for stronger evidence-based critical care.

## CONCLUSIONS

The POETIC framework advances process evaluation in critical care by offering clear dimensions and a tailored structure that address longstanding methodological gaps. It provides a practical foundation for assessing trial and intervention implementation and delivery, supporting more rigorous evaluations and better trial design. As adoption widens across ICU and non-ICU settings, its broader applicability will become clearer, informing refinement and wider use.

## ACKNOWLEDGMENTS

The authors gratefully acknowledge funding from the Medical Research Council (MRC), which supported this work through a 4-year doctoral fellowship as part of the All-Ireland Hub of the MRC Network of Hubs for Trials Methodology Research.

## Supplementary Material
