# Supplementary material for: The POETIC (PrOcess Evaluation of Trials In Critical care) Framework: A Structured Approach for Designing and Conducting Process Evaluations in Critical Care Trials
Source: Crit Care Explor. 2025 Dec 2;7(12):e1355. doi: 10.1097/CCE.0000000000001355 (PMC12674143; doi:10.1097/CCE.0000000000001355)
Supplement: Supplementary file 1 [file cc9-7-e1355-s001.pdf]

<u>Supplementary Material</u>

Table of Contents

Search Strategy for Review 1 2015 1

Flow Diagram for Review 1 2015 2

Search Strategy for Review 1 2025 2

Flow Diagram for Review 1 2025 3

Review 1 Data Extraction Form 4

Search Strategy for Review 2 2016 5

Flow Diagram for Review 2 2016 6

Search Strategy for Review 2 2025 7

Flow Diagram for Review 2 2025 8

Review 2 Data Extraction Form 9

Table S1 Suggested methods and data sources for POETIC Framework dimensions 11

Search Strategy for Review 1 2015

Review 1 search strategy: Ovid MEDLINE (1946 to October 2015)

1. process evaluation.ti.
2. process evaluations.ti.
3. framework.ti.
4. frameworks.ti.
5. guideline.ti.
6. guidelines.ti.
7. guidance.ti.
8. 1 or 2
9. 3 or 4
10. 5 or 6
11. 7 or 9 or 10
12. 8 and 11

Review 1 search strategy: EMBASE (1974 to October 2015)

1. process evaluation.ti.
2. process evaluations.ti.
3. framework.ti.
4. frameworks.ti.
5. guideline.ti.
6. guidelines.ti.
7. guidance.ti.
8. 1 or 2
9. 3 or 4
10. 5 or 6
11. 7 or 9 or 10
12. 8 and 11

Flow Diagram for Review 1 2015

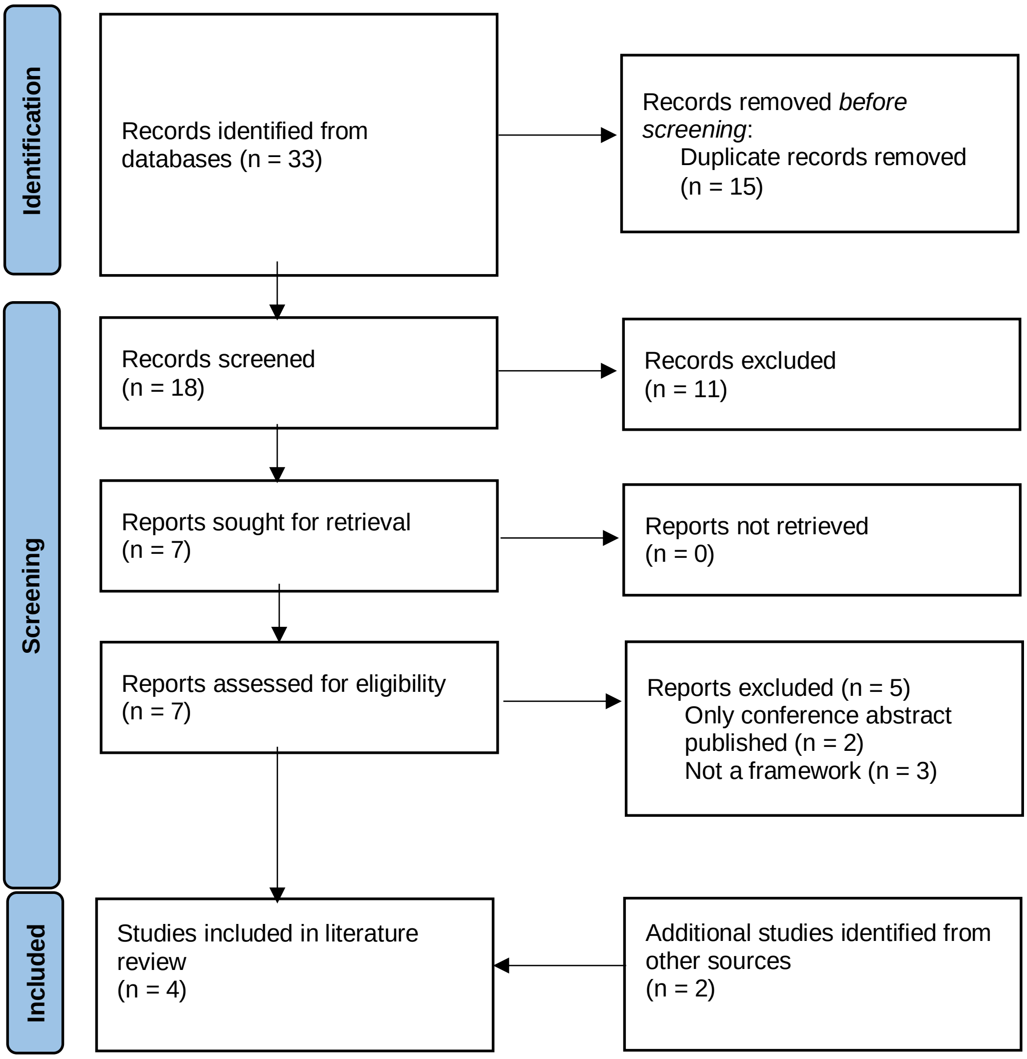

Search Strategy for Review 1 2025

Review 1 search strategy: Ovid MEDLINE (1946 to July 2025)

1. process evaluation.ti.
2. process evaluations.ti.
3. framework.ti.
4. frameworks.ti.
5. guideline.ti.
6. guidelines.ti.
7. guidance.ti.
8. 1 or 2
9. 3 or 4
10. 5 or 6
11. 7 or 9 or 10
12. 8 and 11

Review 1 search strategy: EMBASE (1974 to July 2025)

1. process evaluation.ti.
2. process evaluations.ti.
3. framework.ti.
4. frameworks.ti.
5. guideline.ti.
6. guidelines.ti.
7. guidance.ti.
8. 1 or 2
9. 3 or 4
10. 5 or 6
11. 7 or 9 or 10
12. 8 and 11

Flow Diagram for Review 1 2025

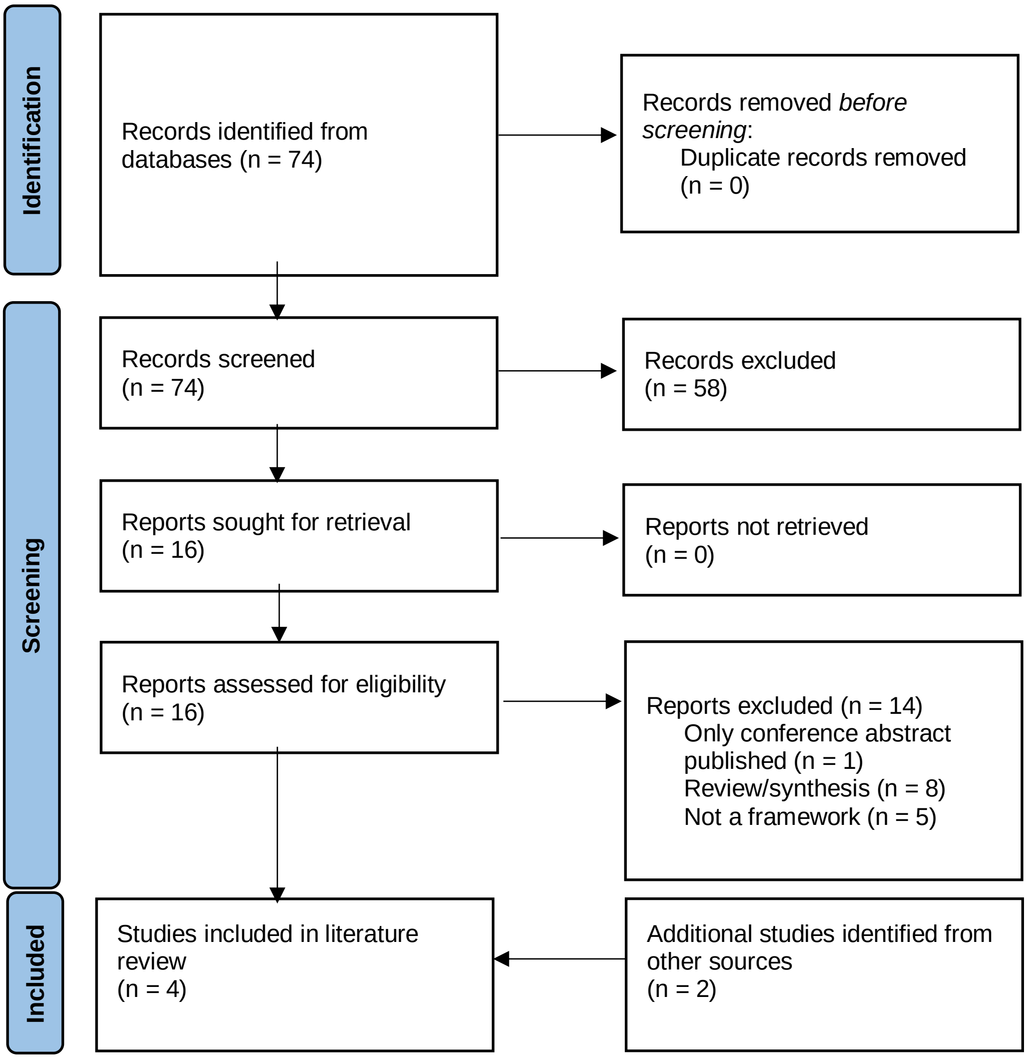

Review 1 Data Extraction Form

**REVIEW 1: PROCESS EVALUATION FRAMEWORKS**

Author			
Title			
STUDY ELIGIBILITY			
1. Does this study relate specifically to a process evaluation framework, guideline, or guidance?		Yes, No, Unclear (specify)	
2. Does the study outcome provide a framework, guidance, or guideline that can be used to guide the future conduct of process evaluation?		Yes, No, Unclear (specify)	
CONCLUSION			
Do not proceed if any of the above answers are 'No'.		Included, Excluded, More information needed (specify)	
DATA EXTRACTION			
1. The context for which the framework was developed			
2. Framework content/dimensions			
3. Dimensions which may be relevant for use in critical care trials			
4. Authors interpretations of strengths and limitations (if reported)			

Search Strategy for Review 2 2016

Review 2 search strategy: Ovid MEDLINE (1946 to December 2016)

1. critical care.ti.
2. critical care.ab.
3. intensive care.ti.
4. intensive care.ab.
5. 1 or 2
6. 3 or 4
7. 5 or 6
8. process evaluation.ti.
9. process evaluation.ab.
10. process evaluations.ti.
11. process evaluations.ab.
12. 8 or 9 or 10 or 11
13. 7 and 12

Review 2 search strategy: EMBASE (1974 to December 2016)

1. critical care.ti.
2. critical care.ab.
3. intensive care.ti.
4. intensive care.ab.
5. 1 or 2
6. 3 or 4
7. 5 or 6
8. process evaluation.ti.
9. process evaluation.ab.
10. process evaluations.ti.
11. process evaluations.ab.
12. 8 or 9 or 10 or 11
13. 7 and 12

Flow Diagram for Review 2 2016

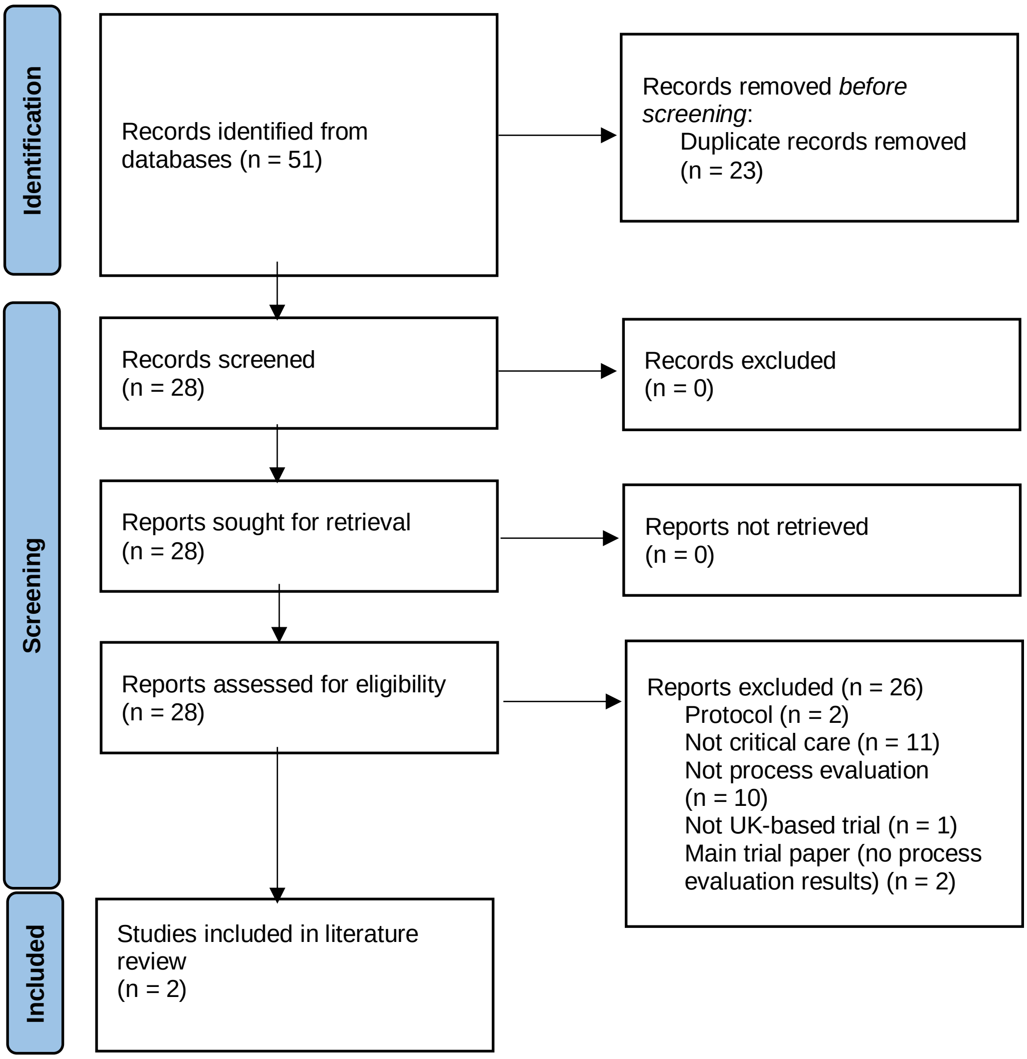

<u>Search Strategy for Review 2 2025</u>

Review 2 search strategy: Ovid MEDLINE (1946 to July 2025)

1. critical care.ti.

2. critical care.ab.
3. intensive care.ti.
4. intensive care.ab.
5. 1 or 2
6. 3 or 4
7. 5 or 6
8. process evaluation.ti.
9. process evaluation.ab.
10. process evaluations.ti.
11. process evaluations.ab.
12. 8 or 9 or 10 or 11
13. 7 and 12

Review 2 search strategy: EMBASE (1974 to July 2025)

1. critical care.ti.
2. critical care.ab.
3. intensive care.ti.
4. intensive care.ab.
5. 1 or 2
6. 3 or 4
7. 5 or 6
8. process evaluation.ti.
9. process evaluation.ab.
10. process evaluations.ti.
11. process evaluations.ab.
12. 8 or 9 or 10 or 11
13. 7 and 12

<u>Flow Diagram for Review 2 2025</u>

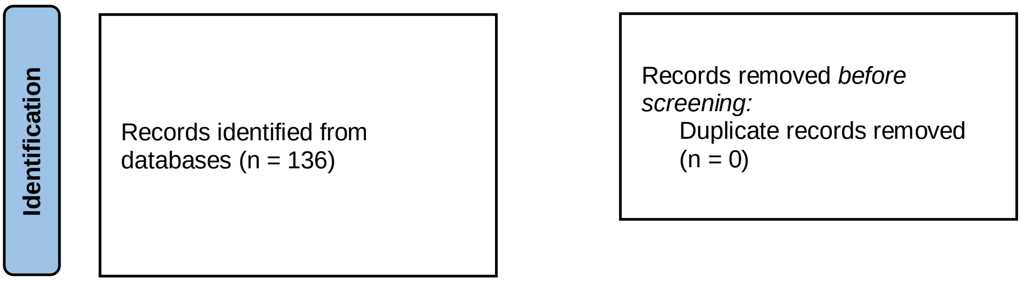

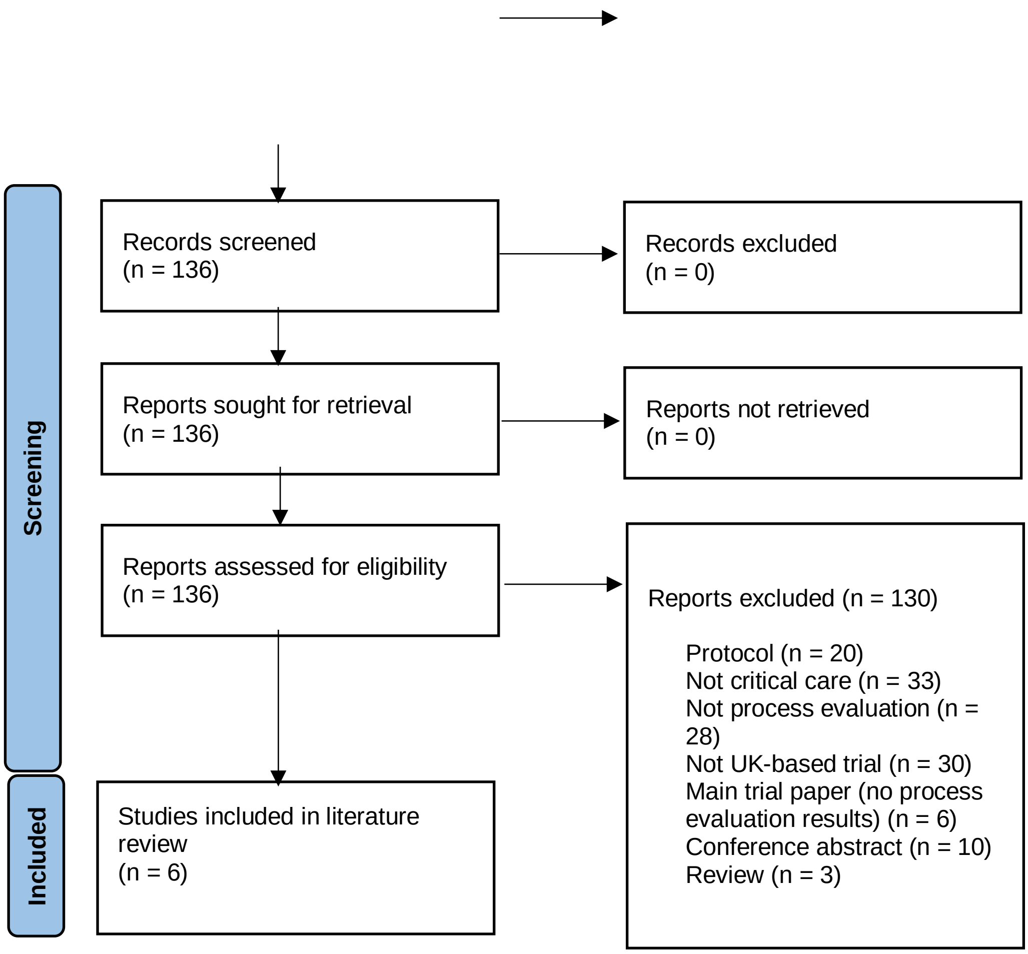

Review 2 Data Extraction Form

**REVIEW 2: PROCESS EVALUATIONS IN CRITICAL CARE TRIALS**	
Author	
Title	
STUDY ELIGIBILITY	

1. Does this study relate specifically to a critical care trial?	Yes, No, Unclear (specify)	
2. Does the study explicitly report incorporating a process evaluation in its design?	Yes, No, Unclear (specify)	
CONCLUSION		
Do not proceed if any of the above answers are ‘No’.	Included, Excluded, More information needed (specify)	
DATA EXTRACTION		
1. Study and design		
2. Process evaluation design/framework		
3. Process evaluation findings/relevance		
4. Authors interpretations of strengths and limitations (if reported)		

Table S1 Suggested methods and data sources for POETIC Framework dimensions.
This table outlines recommended mixed method approaches for assessing each POETIC dimension to support systematic process evaluation.

Framework Dimension	Trial or Intervention Process	Organizational or Participant-Related Factor	Explanatory Notes and Considerations	Suggested Data Sources and Example Questions / Operational Thresholds
How and why delivery was (not) achieved				
**CONTEXT**	Both	Both	An exploration of both organizational and participant-related factors to understand characteristics of the unit in which the trial and intervention are expected to function.<br>Contextual data help to understand in which types of units the intervention works best, and under what circumstances.	See Context sub-constructs 1-5 below.
**1. Unit Culture**	Trial	Organizational	*Consider:*<br>Who takes responsibility/ownership of the trial at units?<br>Is there a permanent trial champion?<br>⇒ How does this impact upon recruitment?<br><br>Does someone disseminate trial-related information to the team?<br>⇒ How does this impact upon study awareness and engagement?	Participant interviews/focus groups<br>Surveys/questionnaires<br><br>**<u>Example question</u>**<br>*"How do staff get to know about new research studies in your unit, and how is information cascaded through the team?"*
**2. Organizational Structure**	Trial	Organizational	*Consider:*<br>Role and clinical experience of the research nurse<br>⇒ How does this affect clinical nurse engagement and participation in research?<br><br>Clinical role and availability of the site PI	Participant interviews/focus groups<br>Surveys/questionnaires<br><br>**<u>Example question</u>**<br>*"Can you tell me who is involved when research is being delivered at the bedside, and what your involvement as nurse is?"*

			⇒ How does this impact study awareness and engagement amongst both clinical and research teams?	
**3. Resources**	Both	Organizational	*Consider:*<br>Is there adequate research nurse provision to deliver the trial?<br>Are there sufficient skilled staff and practical resources to deliver the intervention?<br>Did units implement strategies to overcome resource-related barriers?	Participant interviews/focus groups<br>Surveys/questionnaires<br><br>**Example question**<br>*"Were there times that you couldn't deliver this study because you were under-resourced, and did you manage to find a way to overcome this barrier?"*
**4. Usual Practice**	Intervention	Both	*Consider:*<br>Is usual practice driven by policies/guidelines, unit-consensus, or clinician preference?<br>Usual practice relating to the target intervention problem<br>⇒ How different is the intervention to what is usually done?<br>⇒ Does it challenge deeply entrenched practices?<br><br>Does the intervention require change at participant-level or organizational-level?	Participant interviews/focus groups<br>Surveys/questionnaires<br>Documents/policies/guidelines<br>Observation<br><br>**Example question**<br>*"Can you tell me how you normally manage this practice, and how similar or different is this to what you will be asked to do during the trial?"*
**5. Attitudes and Perceptions**	Intervention	Participant	*Consider:*<br>What beliefs do participants hold about the intervention?<br>⇒ Does this relate to perceptions of risk or benefit?<br>⇒ What impact does usual practice have?<br>⇒ Variation by profession/grade	Participant interviews/focus groups<br>Surveys/questionnaires<br>Observation<br><br>**Example questions**

			How do participants' attitudes and perceptions affect engagement with, and delivery of, the intervention?<br>Intervention recipient<br>⇒ Sedated/ventilated patients versus awake and oriented<br><br>Equipoise, and whether it exists at unit or individual level	*"Can you tell me what attitudes are like toward the trial, and if you think staff view it as worthwhile?"*<br><br>*"Do you or any of your colleagues have safety concerns or anxieties whilst looking after a patient in the trial?"*<br><br>*"Is there equipoise on this topic within your unit?"*
What was achieved?				
**FIDELITY**	Intervention	Both	Evaluate the extent to which the intervention is delivered as intended; and uncover *if, how, and why* intervention fidelity is optimized.	Participant interviews/focus groups<br>Surveys/questionnaires<br>Observation<br>Documentary analysis<br>Protocol compliance/deviation<br><br>**Example question**<br>*"Were there times you couldn't deliver the intervention in accordance with the protocol, and what acted as a barrier?"*<br><br>**Example operational threshold**<br>*≥80% of core intervention steps delivered as intended*

**DOSE**	Intervention	Both	Evaluate the amount of the intervention that is delivered; and uncover *if, how, and why* intervention dose is optimized.	Participant interviews/focus groups<br>Surveys/questionnaires<br>Observation<br>Documentary analysis<br>Protocol compliance/deviation<br><br>**Example question**<br>*"Were there times you couldn't deliver all component parts of the intervention, and why?"*<br><br>**Example operational threshold**<br>*Achieving ≥75% of planned intervention sessions or activities delivered*
**REACH**	Intervention	Both	Evaluate the proportion of intended recipients who received the intervention; and uncover *if, how, and why* intervention reach is optimized.<br><br>Evaluate recruitment rates within units; understand procedures used to ensure/promote recruitment; and explore *if, how, and why* recruitment varies.	Participant interviews/focus groups<br>Trial screening and recruitment logs<br><br>**Example questions**<br>*"In your opinion, were all eligible patients recruited into the study?"*<br><br>*"Did you use any strategies to ensure/promote recruitment?"*<br><br>**Example operational threshold**<br>*≥70% of eligible patients receiving the intervention*
Did it impact upon trial outcomes?				

**QUALITY OF DELIVERY**	Intervention	Both	Developing a composite score permits ranking or grading of units to explore the variation in Quality of Delivery within multi-center trials. The Quality of Delivery scoring algorithm should be developed in conjunction with the process evaluator, intervention developers, oversight committees, and wider co-applicant and trial management groups.<br>To minimize risk of bias Quality of Delivery scoring criteria should be specified *a priori*, be transparent, and available for peer review at all stages of application and publication/reporting.<br>This composite measure also supports explanatory analysis by identifying how variation in delivery quality may contribute to differences in trial outcomes across sites.	A quantitative composite measure of fidelity, dose, and reach.<br><br>**Example operational threshold**<br>*≥80% overall Quality of Delivery index, combining Fidelity, Dose, and Reach*
